# Neuropsychological Health in Mixed Martial Arts Fighters and Combat Sport Athletes: A Scoping Review

**DOI:** 10.7759/cureus.113414

**Published:** 2026-07-26

**Authors:** Lauren Stern, Gabriel J Sanders, Corey A Peacock, Jose Antonio

**Affiliations:** 1 Osteopathic Medicine, Dr. Kiran C. Patel College of Osteopathic Medicine, Nova Southeastern University, Davie, USA; 2 Exercise Science, University of Cincinnati, Cincinnati, USA; 3 Health and Human Performance, Dr. Kiran C. Patel College of Osteopathic Medicine, Nova Southeastern University, Davie, USA

**Keywords:** athletes, combat sports, mixed martial arts, neuropsychological health, repetitive head impacts, traumatic brain injury

## Abstract

Despite safety modifications in mixed martial arts (MMA), traumatic brain injuries remain a significant concern across combat sports due to repetitive head impacts sustained during training and competition. While acute physical effects are well described, long-term cognitive, structural, and psychological consequences of repetitive head trauma in MMA, boxing, kickboxing, wrestling, and other combat sports remain understudied. This scoping review aimed to map the existing literature evaluating these consequences in combat sport athletes. A structured search of PubMed, Scopus, and Web of Science was conducted in January 2026 for studies published between 2020 and 2025. Eligible studies reported neurocognitive, psychological, structural, or biomarker-related outcomes in athletes exposed to repetitive head trauma. Screening was performed by one reviewer, with final studies reviewed by a second investigator for accuracy and consistency. Consistent with scoping review methodology, no formal critical appraisal was conducted, as the aim was to map the breadth of available evidence rather than evaluate methodological rigor. Twelve studies were included. Executive functions, including processing speed, memory, and inhibitory control, were the most consistently impaired domains. Earlier age of first exposure and greater cumulative head impacts were associated with worse outcomes. Structural alterations, including reduced thalamic and hippocampal volume, and increased depressive symptoms were also reported. Overall, current evidence suggests an association between repetitive head trauma and lasting neuropsychological impairment, highlighting the need for improved monitoring and future longitudinal research.

## Introduction and background

Combat sports, including mixed martial arts (MMA), boxing, kickboxing, wrestling, and other disciplines, involve repetitive head impacts that place athletes at risk for traumatic brain injury (TBI). MMA is one of the fastest-growing combat sports, with origins of the sport dating back to around 650 BC [1,2]. It wasn’t until the early 1990s that modern MMA truly emerged with the establishment of the Ultimate Fighting Championship (UFC) [2]. Today, the UFC stands as the leading MMA organization, attracting thousands of fans worldwide, broadcasting to 150 countries, and hosting televised events across 10 nations [1,2].

MMA was initially promoted in North America as a barbaric spectacle designed to determine the most effective unarmed fighting style [3]. With limited regulations and minimal protective equipment, the sport was originally viewed as a brutal practice, with some even referring to it as a "fight to the death” event [3-5]. Modern MMA has since transformed and has adopted a unified set of rules that governs weight classes, refereeing, and judging of events [2,4]. Additional safety measures have also been implemented. Fights are now limited to three to five rounds of five-minute duration each, the use of open-fingered gloves is required to better protect the hands and face, and more harmful techniques such as headbutting have been banned [5]. With these regulations in place, MMA and the UFC have gained broader acceptance and widespread popularity.

Despite these safety modifications, TBIs remain a significant concern amongst MMA fighters and combat sports athletes more broadly. A 2014 systematic meta-analysis of injuries in MMA found that the head was the most commonly injured anatomic region, accounting for 66.8-78.0% of all reported injuries [6]. Another study analyzing all numbered UFC events from 2000 to 2021 found that athletes received an average of 2.41 significant head strikes out of a total of 6.30 head strikes per minute, with head trauma being the leading cause of knockouts [6,7]. Concussions, in particular, are a major concern in all combat sports and often go undiagnosed during competition. In a study that performed detailed video analysis on 60 professional fights, 30 boxing and 30 MMA, it was found that 47 of the matches involved at least one concussion, and in 98% of these cases, the fighter who sustained the first concussion ultimately lost the match [8]. The study also reported that 24 of the 60 fights should have been stopped earlier, indicating a potential need for increased ringside surveillance [8]. Understanding the long-term consequences of repetitive head strikes and knockouts is critical for athlete safety and for determining whether additional protective measures are warranted; however, prior research has primarily focused on the acute effects of head injuries rather than their long-term cognitive, structural, and psychological consequences, highlighting an important gap in the literature.

Given the substantial evidence demonstrating the prevalence of head injuries and concussions amongst combat sport athletes, particularly those participating in MMA, evaluating brain health, mental health, and personality profiling are all critical components to understand in this population. While research specifically focused on MMA athletes is limited, prior literature in athletes more broadly has demonstrated associations between concussion (mild TBI) and mental health outcomes. A 2018 systematic review by Hutchison et al. reported a link between concussion history and depression among former athletes [9]. Evidence from studies of TBIs across varying severities has also identified personality changes, including impulsivity, severe irritability, and aggression, as potential side effects of these injuries, with some TBI patients showing increased suicidality or new-onset psychiatric disorder, including post-traumatic stress disorder (PTSD) [9,10].

Personality traits may influence exposure to head trauma through behavioral risk-taking, as well as willingness to report symptoms, injury recognition, and recovery behaviors. Thus, they provide a relevant framework for understanding variability in neuropsychological outcomes among athletes. Personality profiling has also emerged as a relevant consideration in contact and combat sports, as some research suggests that traits such as risk-taking, impulsivity, and lack of perseverance are more pronounced in athletes who engage in high-intensity or high-impact activities and may be strong predictors of extreme sports injury [11,12]. Sensation-seeking, or the need for novel and exciting experiences, has also been linked, in a study on National Collegiate Athletic Association (NCAA) athletes, to participation in contact sports compared to low- or no-contact sports, as well as to history or incidence of sport-related concussions [13]. These traits may influence both participation in high-impact sports and the willingness to report injury-related symptoms.

Despite these findings, existing reviews have largely examined these domains in isolation, with prior reviews primarily focusing on concussion-related outcomes or structural brain changes without integrating cognitive and psychological dimensions [14,15]. As a result, there remains a gap in the literature examining how these factors interact and impact combat sport athletes exposed to repetitive head trauma. This review addresses this gap by evaluating neuropsychological health in combat sports athletes, with a focus on cognitive outcomes of head trauma, broader psychological health challenges, and personality characteristics. By integrating physiological and psychological perspectives, this paper aims to highlight the unique risks these athletes face as well as opportunities to better support overall athlete health in combat sports.

Specifically, this review addresses the following question: What neurocognitive and neuropsychiatric outcomes and associated structural brain changes or biomarkers have been reported in combat sport athletes exposed to repetitive head trauma? Given the heterogeneity in study designs, outcome measures, and study populations, a scoping review approach was selected to map and synthesize the available literature in this emerging area.

## Review

Methods

Search Strategy and Eligibility Criteria

**Figure 1:**
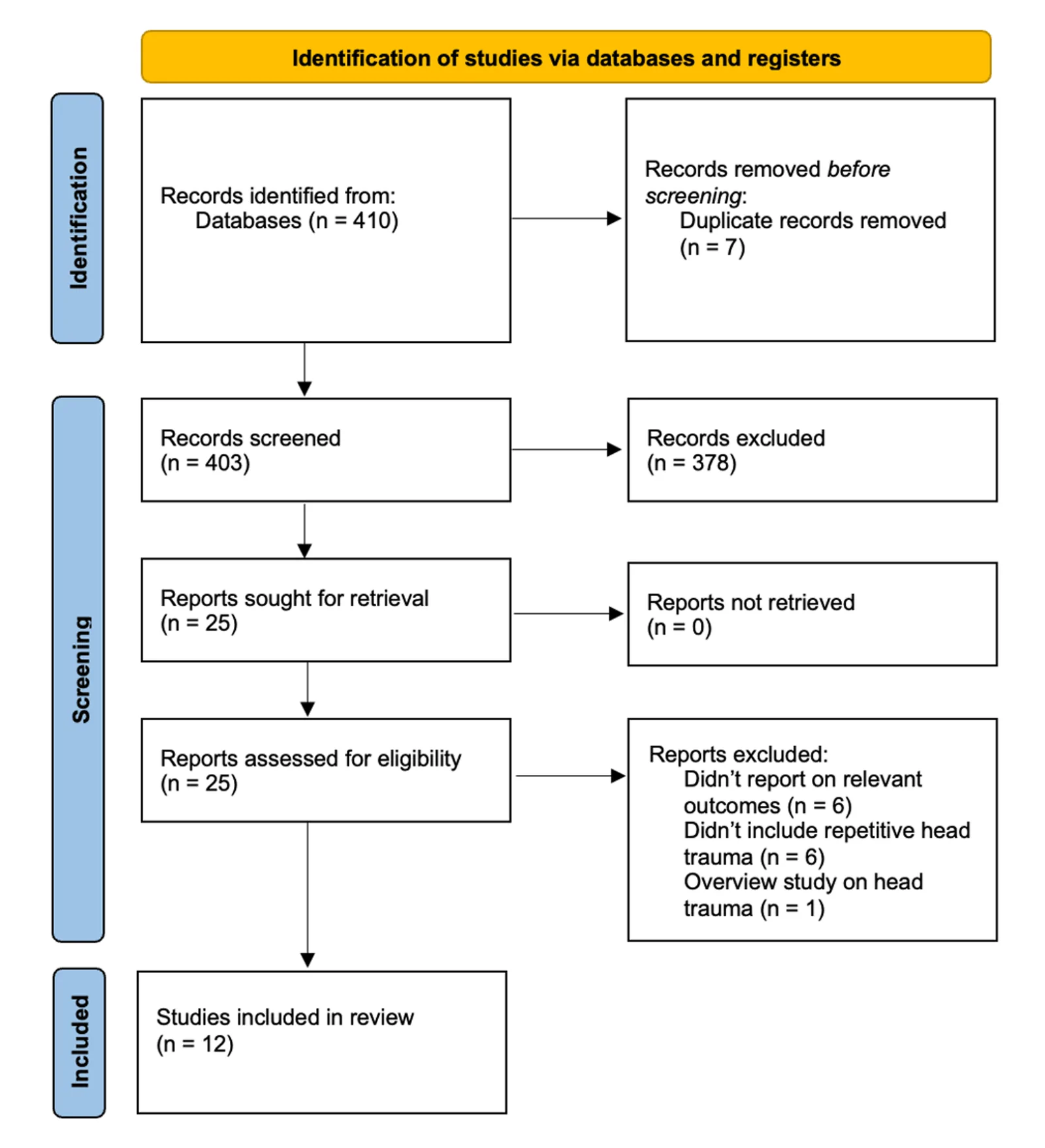
PRISMA-ScR Flow Diagram of Study Selection Process PRISMA-ScR: Preferred Reporting Items for Systematic Reviews and Meta-Analyses Extension for Scoping Reviews

A comprehensive literature search was conducted to identify research examining the neuropsychological consequences of repetitive head trauma in combat sport athletes. This scoping review was conducted in accordance with the Preferred Reporting Items for Systematic Reviews and Meta-Analyses Extension for Scoping Reviews (PRISMA-ScR) guidelines, and a summary of the study selection process is presented in Figure 1 [16]. 

The literature search was conducted in January 2026 across multiple electronic databases, including PubMed, Scopus, and Web of Science. The search strategy combined athlete population and neurocognitive injury keywords using Boolean operators. The PubMed search string used was ("combat sport" OR "mixed martial arts" OR MMA OR UFC OR kickboxing OR boxing OR wrestling) AND ("head injury" OR "traumatic brain injury" OR "brain trauma" OR concussion OR "mild traumatic brain injury" OR TBI OR mTBI OR "cerebral concussion" OR knockout OR neurotrauma OR "brain damage" OR "cognitive impairment"). Filters were applied to include studies published in English between January 2020 and December 2025. Reference lists of included studies and relevant systematic reviews were hand-searched to identify additional eligible studies.

Studies were then assessed according to the following: inclusion criteria comprised (1) studies involving competitive or professional combat sport athletes (e.g., MMA, boxing, kickboxing, wrestling); (2) studies examining exposure to repetitive head impacts, concussion, or TBI; and (3) studies reporting neurocognitive, psychological, structural brain, or biomarker-related outcomes. Exclusion criteria comprised (1) studies not involving combat sport populations; and (2) studies not assessing head trauma or related outcomes. Studies outside the predefined search limits (English language and publication between January 2020 and December 2025) were also excluded.

Study Selection and Data Extraction

Screening of titles and abstracts was conducted by one reviewer using predefined inclusion and exclusion criteria, with all potentially eligible full-text articles independently reviewed by a second investigator to ensure accuracy and consistency. Data extraction was performed by one reviewer using a standardized form and subsequently verified by a second investigator through cross-checking of all extracted variables. Discrepancies were resolved through discussion and consensus, with reviewers revisiting the predefined inclusion and exclusion criteria to reach agreement. If agreement could not be met, the article was not included. Extracted variables included study design, sample size, participant demographics (e.g., age and sex), type of combat sport, exposure characteristics (e.g., training intensity and fight history), and outcomes of interest, including cognitive measures, structural brain metrics, and relevant biomarkers. Where available, statistical measures, including p-values, effect estimates, and confidence intervals, were also reported. Only explicitly reported data were used, and no assumptions were made regarding missing or unclear data.

In total, 410 articles were identified through searches of PubMed, Scopus, and Web of Science. After removal of duplicates (n = 7), 403 records underwent title and abstract screening, of which 25 articles were retained for full-text review. Of these, six articles were excluded because they did not report relevant outcomes, six were excluded because the cohorts did not experience repetitive head trauma, and one was excluded because it was a narrative overview that did not report specific findings. Twelve evidence sources were therefore deemed eligible for inclusion in the review (Figure 1). To minimize duplication of evidence, previously published reviews were included for contextual interpretation only and were not treated as independent sources of primary data in the synthesis.

Among the included evidence sources, there was one narrative review, two systematic reviews, and nine primary research articles, including cross-sectional, longitudinal cohort, case-control, and pilot observational studies published between 2020 and 2025. This time frame was selected in part because of a prior comprehensive review published in 2021 that evaluated literature regarding cognitive decline in MMA fighters exposed to head injuries and/or technical knockout (TKO) or knockout (KO) events through May 2020 [17]. Given the continued growth and popularity of MMA and other combat sports, the present review aims to build upon existing work by incorporating more recent evidence while minimizing duplication of previously synthesized findings.

Given the heterogeneity of included studies in terms of design, populations, and outcome measures, a qualitative synthesis approach was used, and studies were grouped based on outcome domains, including cognitive function, structural brain changes, and biomarker-related findings. Potential sources of heterogeneity, including differences in study design, participant characteristics, and exposure levels, were explored descriptively. Consistent with scoping review methodology, formal risk of bias, reporting bias, and certainty of evidence assessments were not conducted, as the objective was to map the breadth of available evidence rather than critically appraise study quality. Although duplicate independent screening and extraction were not fully implemented, the use of predefined criteria and secondary verification was intended to enhance consistency and reduce potential error. Primary research articles were the main unit of analysis, while included reviews were used for contextual interpretation only.

Neuropsychological health in combat sports

Cognitive Health

Given the prevalence of concussions and head trauma in MMA fighters and other combat sport athletes, assessing the long-term impact of these injuries on cognitive function is important to ensure athlete safety. Head trauma can cause both short- and long-term consequences depending on the severity of the injury [18]. Some of the most common clinical changes resulting from head trauma include headaches, blurred vision, dizziness, increased sensitivity to light and noise, sleep disturbances, decreased reaction time, sudden mood swings, and memory problems [14]. Repetitive head trauma over time can even lead to a progressive neurodegenerative disease called chronic traumatic encephalopathy (CTE). CTE results in a further decline in memory and cognition, along with behavioral and mood changes such as depression, poor impulse control, aggressiveness, and parkinsonism [19,20]. Due to limitations in diagnosing CTE, traumatic encephalopathy syndrome (TES) has been proposed as the clinical presentation of suspected CTE [21]. Long-term consequences of sports-related concussions (SRC) have also been associated with increased suicide attempts [18,20]. Given the high-impact nature of MMA and other combat sports, along with the frequency of head trauma, research focused on determining the most effective methods to identify concussions and injuries as early as possible is essential to minimize long-term consequences in processing speed, verbal memory, and psychomotor speed [17].

One measure frequently used to assess cognitive function is the use of blood biomarkers. The first, glial fibrillary acidic protein (GFAP), is found in blood plasma and is a marker of astrocytic injury or activation [22]. While currently being assessed as a potential biomarker for detecting Alzheimer’s disease, GFAP levels can also be used as an indicator of TBI [18,23,24]. Studies have shown that GFAP levels can be detected in blood less than one hour after a head injury and is accurate in detecting mild to moderate TBI, intracranial lesions on head computed tomography (CT), and the need for neurosurgical intervention [24]. Another promising blood biomarker is ubiquitin C-terminal hydrolase (UCH-L1), which is a marker of neuronal injury present in patients with TBI of all categories, and expression may be associated with severity of injury [18]. Brain-derived neurotrophic factor (BDNF), an autocrine that supports neuron development and survival, has also demonstrated a strong association with TBI [18]. With further research, these biomarkers can be useful to assess risk, predict acute or long-term outcomes, and support decision-making for professional fighters.

Aside from blood biomarkers, additional tests are available and are widely utilized to assess cognitive impairment. One commonly used exam for detecting SRCs in sports medicine is the Sport Concussion Assessment Tool (SCAT), which is now in its sixth version released in 2023 [25]. SCAT6 is a multi-component assessment that can be administered on the sideline and incorporates several elements such as red flag symptoms, balance/postural control, Glasgow Coma Scale, cervical spine assessment, and memory assessment to assist in clinical diagnosis. Research has found that SCAT test results are most accurate within the first 72 hours, with clinical accuracy decreasing up to seven days post-injury [25,26]. Although comprehensive, a proper SCAT6 evaluation can take 10-15 minutes to complete, which often makes it impractical as the first choice for trainers and physicians to use in time-restricted scenarios, such as between rounds in an MMA match [27]. Instead, a ringside exam that can be used to assess subtle changes in vision, attention, and processing speeds is the King-Devick (K-D) test, which can be administered in as little as two minutes [27]. In a 2019 study by Hubbard et al., K-D times significantly worsened in the majority of athletes following an ‘event’, defined as a KO, TKO, choke, or submission. In comparison, during standard workouts or matches without an ‘event’, the K-D time improved, suggesting that this test can effectively identify concussions or brain injuries in athletes [28].

Neuroimaging techniques have also become an important tool for assessing head trauma and potentially predicting cognitive decline. While CT remains the first-line imaging modality, more advanced techniques such as T2-weighted magnetic resonance imaging (MRI), fluid-attenuated inversion recovery (FLAIR) MRI, and diffusion-weighted imaging (DWI) are more sensitive to traumatic lesions and may be better able to assess brain structure and integrity of white matter tracts [29]. Research continues to assess whether measures such as brain volumes and atrophy can serve as predictors of TES and cognitive decline [21]. Thus far, neuroimaging seems like a promising tool to provide a more comprehensive assessment of the extent and severity of the brain injury.

Overall, the growing use of blood biomarkers, rapid sideline evaluations, and neuroimaging reflects an important shift toward earlier and more precise identification of brain injuries in fighters. As these methods continue to advance, they can help clinicians better recognize head trauma during competition and reduce the risk of long-term neurological decline.

Mental Health Disorders

Aside from cognitive decline, TBIs can also significantly affect psychological well-being and increase the risk of developing mental health disorders. Historically, elite athletes have been less likely to report mental health symptoms or seek treatment, largely due to stigma, low mental health literacy, and fear that disclosure may negatively impact their standing within their sport [30]. Although research focused on mental health in MMA fighters is limited, a 2025 study examining 45 current and 29 former elite kickboxers found notably high rates of mental health symptoms. Among current athletes, the most common issues were psychological distress (57%) and disordered eating (63%), while former athletes most frequently reported psychological distress (36%) and alcohol misuse (43%) [31]. Given both the prevalence of mental health concerns in elite athletes and the ongoing stigma surrounding mental health disclosure, understanding how brain injuries influence psychological health is especially crucial in this population.

The high rate of alcohol misuse among former athletes highlights the need to better understand the factors driving these behaviors. Alcohol and substance use have been reported in both current and former athlete populations for reasons such as socialization, self-medication for pain or anxiety, and sleep facilitation [32,33]. Moreover, as athletes transition out of competitive sport, alcohol use can also increase as individuals struggle with loss of identity, chronic pain, or persistent psychological distress [33]. Although research remains limited, it is important to consider the effect of TBIs in exacerbating vulnerability to maladaptive coping mechanisms, including substance abuse.

In addition to substance abuse, TBIs can also significantly impair emotional regulation, making individuals more vulnerable to developing anxiety and depression. TBIs are characterized by structural alterations in the brain and involve both primary and secondary injury mechanisms. Primary brain injury occurs at the moment of impact and results from disruption of the neuronal cell membrane, whereas secondary brain injuries develop over time due to excitatory toxicity, mitochondrial dysfunction, oxidative stress, lipid peroxidation, and neuroinflammation [34]. The specific neuropsychiatric consequences often depend on the brain regions affected, most commonly the prefrontal cortex, amygdala, and hippocampus, which play critical roles in mood regulation, stress response, and reward processing [34,35]. From a pathophysiological perspective, the cellular response to injury is largely dependent on oxygen radical reactions, excitatory amino acid release, and nitric oxide production, all of which contribute to cell depolarization and potentially cell death [36]. Collectively, these structural and biochemical disruptions may impair neural circuits involved in emotional and cognitive processing, thereby increasing the risk of long-term psychiatric disorders, such as depression, anxiety, and PTSD.

Symptoms of depression can also adversely affect recovery following concussion. In a study that compared patients post-TBI who self-reported symptoms of depression to patients post-TBI without depression, researchers found that those with depression reported more post-concussion symptoms and higher overall symptom severity [37]. These findings add to the growing evidence of a complex bidirectional relationship between mood symptoms and concussion recovery, and more broadly, injury recovery in athletes. SRC are associated with worse mental health and quality of life, while pre-existing or concurrent mental health symptoms are, in turn, linked to slower and more prolonged recovery trajectories [38]. Given this relationship, early recognition and treatment of depression is especially important in combat athletes who have sustained a brain injury, as unmanaged mood symptoms can slow neurological healing and prolong overall recovery.

PTSD is another cognitive complication that remains underrecognized in combat sport athletes and may have an association with TBIs. While difficult to determine the exact prevalence due to underreporting, it is estimated that one in eight elite athletes suffer from PTSD [39]. The epidemiology of PTSD specifically in combat sport athletes has not yet been well studied; however, given the violent and aggressive nature of these sports and the documented prevalence of PTSD in elite athletes, it is reasonable to hypothesize that PTSD is also present in this population. In relation to TBIs, a study of collegiate and semi-professional athletes found that symptom scores of PTSD were greater in post-concussed athletes compared to healthy controls, with the most reported symptoms being “difficulty sleeping”, “avoiding similar situations”, “having trouble keeping thoughts of incident out of your head”, and “flashbacks” [40]. Given the frequency of traumatic exposure in MMA fighting, including KOs and severe physical injury, along with the high rate of repeated head injuries, these factors may further contribute to the development or persistence of PTSD symptoms in this group.

Personality Profiles

Previous research has explored the relationship between personality profiles and sports participation, but the literature in this area remains limited. There are several approaches to personality profiling, with some of the most commonly used being the Big Five and Myers-Briggs Type Indicator (MBTI).

The Big Five personality traits consist of neuroticism, extraversion, openness, agreeableness, and conscientiousness [41]. Researchers have also explored how these traits may relate to other characteristics such as resilience, risk-taking, and aggression, all traits which are commonly observed in athletes participating in high-risk sports [41-43]. For example, athletes who engage in higher levels of risk-taking were found to exhibit a personality profile characterized by low conscientiousness in combination with either high neuroticism or high extraversion [43]. In a separate study focusing on aggressive behavior, researchers found that openness, agreeableness, and neuroticism were all directly and indirectly associated with physical aggression, while conscientiousness showed no relation with aggression or violence [42]. Resilience is another important trait in athletes. Ego-resiliency, or an individual’s dynamic ability to adjust reactions and perceptions to meet situational demands, has been shown to have a stronger negative relationship with neuroticism and a stronger positive relationship with openness and agreeableness [41].

The MBTI is another personality profiling questionnaire that categorizes individuals into one of 16 possible personality types. These personality types are generated from main dimensions: introversion or extraversion, sensing or intuition, thinking or feeling, and judging or perceiving [44]. Research on the influence of MBTI personality types on athlete personality and adaptability remains limited, but one study found that extreme athletes scored higher in introversion and perceiving compared to traditional sport athletes, and that perceiving was highly correlated with sensation-seeking [45].

While these findings provide foundational insight into athlete personality, their direct implications for head trauma exposure and recovery in combat sport athletes remain largely theoretical. Certain traits, such as sensation-seeking, impulsivity, and high-risk tolerance, may influence engagement in frequent sparring or symptom disclosure following head impacts, whereas resilience and conscientiousness may be associated with adherence to recovery protocols. Although baseline personality profiling in fighters may offer valuable neuropsychological context, its use for identifying individuals at higher risk or informing individualized interventions should be considered a hypothesis requiring further investigation rather than a current clinical application. Future research is needed to better understand how these traits interact with exposure and reporting behaviors, and whether they can meaningfully inform monitoring strategies or targeted approaches to support recovery and resilience.

Results 

Overall Study Findings

The study selection process is summarized in Figure 1. A comprehensive synthesis of the existing literature on the effects of head trauma in combat fighters, including MMA fighters and boxers, has identified numerous consequences affecting both brain structure and executive function. Table 1 consolidates evidence across cognitive, structural, and neuropsychiatric domains in order to illustrate the effects of this trauma. Key statistical findings, including representative effect estimates and p-values where available, are summarized in Table 1.

**Table 1:** Head Trauma and Outcomes in Combat Sports MMA: mixed martial arts; GFAP: glial fibrillary acidic protein; MRI: magnetic resonance imaging; AFE: age of first exposure; TES: traumatic encephalopathy syndrome; MPS: mental processing speed; IC: inhibitory control; CF: cervical flexibility; BDNF: brain derived neurotrophic factor; PLR: pupil light reflex; mTBI: minor traumatic brain injury; NINDS: National Institute of Neurological Disease and Stroke; CTE: chronic traumatic encephalopathy

Study (Year)	Title	Theme	Outcome	Key Statistical Results
Barcelos et al. (2024) [14]	Concussion and executive functions in combat sports: a systematic review	Impact of concussions on core executive functions in combat sport athletes, specifically boxing and MMA.	Approximately 81.9% of the studies reported executive function impairments associated with sports-related brain trauma. The most commonly affected domains were memory (36.5%) and IC (27.5%).	Executive function impairment reported in 81.9% of studies; memory (36.5%) and inhibitory control (27.5%) most affected.
Bernick et al. (2025) [22]	Blood biomarkers and neurodegeneration in individuals exposed to repetitive head impacts	Relationship between blood biomarkers and longitudinal change in cognitive function and brain volume in active and retired MMA fighters and boxers.	Baseline GFAP levels were highest among retired boxers, and longitudinal increases in GFAP were associated with decreased volumes in several cortical and subcortical brain regions, along with enlarged lateral ventricles. Cognitive performance, including memory, processing speed, psychomotor speed, and reaction time, declined over time as GFAP increased.	GFAP increased longitudinally and was associated with reduced brain volumes (e.g. hippocampus: B = -1.25, 95% CI -1.65 to -0.85) and declines in cognitive performance.
Bryant et al. (2020) [46]	The effect of age of first exposure to competitive fighting on cognitive and other neuropsychiatric symptoms and brain volume	Compared active and retired licensed professional fighters (MMA or boxing) to a control group to measure AFE to fighting sports and brain structure, cognitive performance, and clinical neuropsychiatric symptoms.	Brain MRI showed significant correlations between earlier AFE to fighting sports and smaller bilateral hippocampal and posterior corpus callosum volumes in both retired and active fighters. In active fighters, earlier AFE was associated with reduced processing speed and psychomotor speed, while in retired fighters, earlier AFE was linked to higher levels of depression and impulsivity.	Earlier AFE was associated with smaller hippocampal volume (p = 0.005) and reduced processing speed (p < 0.001).
Conway Kleven et al. (2023) [21]	Longitudinal changes in regional brain volumes and cognition of professional fighters with traumatic encephalopathy syndrome	Comparing regional brain volumes and cognitive performance between fighters with TES and without.	In fighters with TES, the rate of cognitive decline was significantly greater for reaction time. Statistically significant interactions and between-group mean differences were also observed across all MRI volumetric measurements, with the TES+ group showing more pronounced structural changes compared with the TES- group.	TES+ fighters showed greater cognitive decline in reaction time (estimate = 56.31, 95% CI 26.17 to 86.45).
de Brito et al. (2025) [47]	Long-term cognitive decline in MMA fighters: a two-year cohort study on executive function impairments due to repetitive head strikes	Executive functions were assessed in a competitive group, consisting of MMA fighters regularly exposed to high-impact training, and a recreational group, composed of MMA fighters with minimal head strike exposure.	The competitive group showed significantly larger declines in MPS, IC, and CF after 1 and 2 years. Additionally, both automatic and controlled processes, as well as direct and indirect memory, were impaired in this group.	Greater declines in processing speed over 2 years (14.6s difference, p = 0.003), inhibitory control (p ≤ 0.001), and cognitive flexibility (p ≤ 0.001).
de Lima et al. (2024) [18]	Levels of biomarkers associated with subconcussive head hits in mixed martial arts fighters	Evaluation of biomarker levels associated with sub-concussive hits to the head.	Significant baseline differences in BDNF levels were observed between active and healthy fighters. Additionally, BDNF levels showed a significant decrease from immediately after the sparring session to 72 hours post-session.	BDNF levels differed at baseline (p = 0.03) and decreased immediately post-sparring to 72 h (p = 0.03).
Holland et al. (2025) [48]	Identifying cervical predictors of recreational mixed martial arts participation: a case-control study	Cervical spine function and self-reported symptoms were compared between MMA fighters and individuals participating in general fitness training.	The MMA group exhibited significantly higher scores for post-concussion symptoms, cervical flexor endurance, and the number of concussions, but showed lower flexion range of motion.	The MMA group showed higher post-concussion symptoms (p = 0.012), cervical flexor endurance (p = 0.031), and concussion frequency (p = 0.001).
Kirk and Childs (2023) [49]	Combat sports as a model for measuring the effects of repeated head impacts on autonomic brain function: a brief report of pilot data	To determine which automated PLR variables are affected by MMA sparring.	Testing revealed decreased maximum and minimum pupil size, and reduced PLR latency, following sparring. Anisocoria was present before sparring and worsened afterward, with both eyes showing differences in minimum and maximum pupil sizes and constriction velocities post-sparring.	Decreased maximum and minimum pupil size and reduced PLR latency post-sparring (BF₁₀ ≈ 3–4).
Merino et al. (2023) [15]	Examining the occurrence and outcomes of concussion and mTBI in mixed martial arts athletes: a systematic review	To assess the occurrence, outcomes, and consequences of mTBI in MMA athletes.	Deficits in memory, reaction time, and processing speed were observed following the occurrence of mTBI.	Greater mTBI occurrence in MMA athletes; associated deficits in memory, reaction time, and processing speed.
Neel et al. (2023) [50]	Articulation rate, pauses, and disfluencies in professional fighters: potential speech biomarkers for repetitive head injury.	Examined articulation rate, pausing, and disfluency in reading passages to assess neurological decline associated with repetitive head injury in professional boxers and MMA fighters.	Boxers and MMA fighters showed differences in articulation rate, producing syllables nearly half a syllable per second slower compared to controls. Additionally, boxers exhibited significantly more pauses and disfluencies compared to both controls and MMA fighters.	Slower articulation rate (~0.5 syllables/sec) in boxers and MMA vs controls. Boxers showed significantly more pauses and disfluencies.
Ritter et al. (2022) [51]	Traumatic encephalopathy syndrome: application of new criteria to a cohort exposed to repetitive head impacts	Tested whether fighters who met recent NINDS consensus diagnostic criteria for TES showed differences in MRI-based regional volumes, cognitive domains, and certain plasma biomarkers.	Of 176 participants (110 boxers and 66 MMA), 72 were classified as having TES based on the newest criteria. Fighters with TES tended to have started fighting at a younger age, had more professional fights, and experienced more frequent knockouts. The TES+ group exhibited lower regional brain volumes, including in the thalamus, hippocampus, posterior corpus collosum, and overall white and gray matter. Additionally, they scored lower on various cognitive measures, including simple and choice reaction time and psychomotor speed.	41% (72/176) classified as TES; TES+ showed reduced regional brain volumes (95% CI -613 to -120 mm^3^) and impaired cognitive performance.
Schlegel et al. (2021) [17]	Head injury in mixed martial arts: a review of epidemiology, affected brain structures and risks of cognitive decline	Systematic literature review on the association between head injuries and cognitive functions in MMA fighters.	Fighters who sustained head trauma have worse post-match King-Devick scores, and impairments in processing speed, verbal memory, and psychomotor speed. Head trauma is also a risk factor for developing neurodegenerative disorders, including potentially CTE.	Head trauma accounted for 58-78% of all injuries; associated with worse post-match King-Devick scores and cognitive impairments.

Cognitive/Executive Function Decline

Multiple studies have examined the effects of brain trauma across various domains of executive function in combat sport athletes. Executive functions are defined as a set of mental processes that are essential for cognitive, psychological, and social development, as well as for academic and professional performance. Deficits are frequently reported, with some studies documenting impairments in over 80% of athletes with repeated head trauma [14]. In the context of MMA, executive function is commonly assessed using measures of cognitive flexibility (the ability to switch between different combat modalities and adjust tactics), processing speed, inhibitory control, and working memory [14,47]. Across the studies included in this review, memory, processing speed, and inhibitory control were the domains most consistently found to decline in fighters.

Processing speed was examined in six of the articles included in this review. Bryant et al. found that an earlier age of first exposure (AFE) to competitive fighting was associated with significantly reduced processing speed [46]. In addition to earlier fighting onset, declines in mental processing speed were also linked to more frequent and intense training where athletes suffered greater head strike exposure [15,47]. Supporting this, de Brito et al. reported that highly competitive fighters exhibited greater impairments in mental processing speed compared to a recreational training group, with a mean difference of 14.6 s (p = 0.003) [47].

Memory decline was assessed in five studies and was identified as another key cognitive domain affected by repeated head trauma in fighters. Barcelos et al. assessed over 1,100 fighters, a mix of boxers and MMA fighters, and found that 81.9% showed executive function impairment, with memory impairment being the most frequently affected at 36.5% [14]. Consistent with processing speed findings, fighters exposed to higher levels of recurrent head impacts showed greater decline in memory [15,17,47]. Impairments in these domains were associated with reduced performance on tasks involving instruction-following, reaction, and strategy execution [47].

Inhibitory control is less frequently studied but remains an important cognitive domain. It is defined as the capacity to suppress impulsive or automatic responses in order to respond appropriately to a situation [14]. Research has shown that fighters with repeated head injuries experience declines in inhibitory control, which have been associated with impaired decision-making performance in both sport-related and non-sport contexts [14,47].

Structural Brain Changes

In addition to executive function, several studies examined structural brain differences associated with repetitive head trauma using neuroimaging techniques. Some of the most consistently affected regions across studies include the thalamus, hippocampus, and posterior corpus callosum (CC). Additional findings have included lateral ventricle enlargement, decreased white matter and both subcortical and total gray matter volume, as well as decreased caudate and putamen volumes [21,22,51].

Focusing first on the thalamus, multiple studies have found that the thalamus is among the brain regions most affected by TBI. Ritter et al. reported that professional fighters with diagnosed TES (TES+) have significantly smaller thalamic volumes compared to those without TES (TES-), with volume differences ranging from -613 to -120 mm3 [51]. Conway Kleven et al. found consistent results showing an estimated mean difference of -784.01 mm3 in TES+ fighters compared to TES-, and additionally identified longitudinal atrophy in the thalamus of active fighters compared to a control group [21]. Although the thalamus naturally decreases in volume with age, the alteration in thalamo-cortical connectivity has been linked to attention disorders, and changes in working memory and executive function [52].

The hippocampus is another brain region that is consistently identified as being affected. This structure plays a critical role in spatial learning and memory consolidation, and reduced hippocampal volume has been strongly linked to depression [53,54]. Using MRI data, Bryant et al. reported that fighters with earlier AFE had smaller bilateral hippocampal volumes in both active and retired athletes [46]. Similarly, when comparing TES+ and TES- fighters, Conway Kleven et al. found an estimated mean hippocampal volume difference of -385.04 mm3 [21]. Reduced hippocampal volume has been associated with depression in prior literature.

Similarly to the thalamus and hippocampus, reductions in posterior CC volume have also been reported in association with repetitive head trauma. When comparing the posterior CC volume in TES+ and TES- fighters, Conway Kleven et al. reported an estimated mean difference of -147.98 mm3, as well as longitudinal atrophy in this region in active fighters compared to controls [21]. Earlier AFE was also significantly correlated to smaller posterior CC volumes in active and retired fighters [46]. The posterior CC is the major connection between the left and right cerebral hemispheres and is crucial for processing sensory, motor, and high-level cognitive signals [55]. Overall, these studies demonstrate consistent regional volumetric differences associated with measures of head impact exposure across combat sport athletes.

Biomarkers and Physiologic Indicators

Two studies included in this review examined the association between biomarkers and repetitive head trauma. The first, by de Lima Filho et al., found significantly different baseline BDNF levels between active fighters and healthy controls, as well as a significant reduction in BDNF levels between immediately post-sparring and 72 hours after the session [18]. The second study by Bernick et al. assessed a cohort of active and retired boxers and MMA fighters and found that baseline GFAP levels were highest in retired boxers [22]. In retired athletes, elevated GFAP levels were associated with decreased cortical and subcortical volumes, increased lateral ventricle size, and lower performance in cognitive domains such as memory, processing speed, and reaction time. Among active fighters, elevated GFAP was correlated with lower thalamic and CC volumes and worsening psychomotor speed [22].

Additional Results of Synthesis

Across the included studies, findings were generally consistent in direction, with impairment most frequently observed in executive function domains and structural brain measures. However, some variability in the magnitude and statistical significance of results was noted, likely reflecting differences in study design, participant characteristics, exposure levels, and outcome measures. Where reported, effect sizes were typically small to moderate, although these were not consistently provided across all studies, thus limiting direct comparison.

The current body of evidence is largely derived from observational and cross-sectional studies. Consistent with the scoping review design, a formal assessment of risk of bias was not conducted, and reporting bias and certainty of evidence were not formally evaluated. Additionally, no sensitivity analysis was performed.

Discussion

Accumulating evidence indicates that combat sport athletes exposed to repetitive head trauma may be at heightened risk for neuropsychological impairment, with both AFE and the intensity or cumulative burden of head impacts appearing to influence outcomes. These findings align with prior research suggesting that earlier exposure to fighting is associated with differences across multiple neuropsychological domains, including reductions in regional brain volumes, slower processing speed, and elevated depression symptoms [14,46]. Together, this evidence suggests that earlier and more frequent head trauma exposure may have implications for long-term cognitive and psychological outcomes in combat sport athletes. Given the scoping review design, these findings should be interpreted as a descriptive synthesis intended to identify patterns, themes, and gaps in the literature rather than provide pooled quantitative estimates or definitive causal conclusions.

While the overall direction of findings was largely consistent, some variation was observed across studies depending on the specific cognitive domain, biomarker, or comparison group. Differences in the magnitude of reported effects were noted, with effect sizes generally appearing small to moderate where described. The current body of literature is primarily composed of observational and cross-sectional studies, and variation in sample characteristics and study design should be considered when interpreting findings. Collectively, these patterns support a consistent association between repetitive head trauma and neuropsychological outcomes, while underscoring the need for larger, longitudinal investigations.

Among these outcomes, executive functions, including processing speed, memory, and inhibitory control, appear to be the most consistently affected cognitive domains, highlighting their susceptibility to impairment. Declines in inhibitory control may have broader implications, as impairments in this domain have been associated with altered decision-making both in athletic settings and everyday life. Similarly, deficits in processing speed and memory may affect sport performance, as deficits in these areas can potentially influence reaction timing, adherence to instructions, and execution of strategies under dynamic conditions.

These functional deficits are paralleled by measurable structural alterations in key brain regions, including volumetric reductions in the thalamus, hippocampus, and posterior CC, regions that are critically involved in attention, memory consolidation, and interhemispheric communication. The presence of such atrophic changes in young fighters may reflect structural patterns that warrant further longitudinal investigation and may be associated with features of early-onset cognitive dysfunction. Notably, reductions in hippocampal volume may contribute to the high rates of depression observed in athletes following a concussion, representing an important target for ongoing monitoring. However, despite the relevance of mental health outcomes such as depression and anxiety, as well as personality-related factors, these domains were not consistently or comprehensively evaluated across the included studies, highlighting an important gap in the literature.

In addition, alterations in biomarker levels, including GFAP and BDNF, provide physiologic evidence consistent with ongoing neural stress and biological changes that may reflect early or evolving neural dysfunction. Supporting this relationship, Bernick et al. found that increased levels of GFAP were associated with decreased cortical and subcortical volumes and enlarged lateral ventricles [22]. Longitudinal measurement of GFAP may help identify those at potential risk for future regional and/or cognitive changes. These findings suggest that repetitive head impacts may be associated with neurobiological changes observed across studies that extend beyond acute injury and may influence long-term neurological health in this population.

Taken together, the convergence of structural brain alterations, biomarker changes, and cognitive deficits suggests that these domains are likely interconnected rather than independent. Blood-based biomarkers such as GFAP and BDNF, both of which have been found at elevated levels in active and retired fighters, have been associated with TBI and may reflect underlying neural stress [18,22]. Studies evaluating elevated GFAP levels have found links to volumetric reductions in brain regions including the thalamus, CC, and cortical and subcortical areas, which in turn have been associated with impairment in executive function, processing speed, and memory [22]. These relationships, however, are not consistently assessed within the same cohort groups and should be considered correlational rather than causal. Combining biomarkers, neuroimaging, and cognitive assessments within a single study offers a promising strategy for future research to better understand how these factors interact and contribute to the neurobiological consequences of repetitive head impacts.

As noted earlier, enhanced rules and safety measurements have been implemented in MMA fighting to improve athlete protection. However, given the findings of this review, it may be reasonable to consider additional medical supervision, both at ringside and longitudinally throughout an athlete’s career in order to better protect cognitive and psychological well-being. Ringside monitoring could include greater implementation of rapid cognitive screening tools, such as the King-Devick test, any time an athlete receives a significant blow to the head, as well as the use of rapid handheld blood tests to assess injury-related biomarkers. Additional policy considerations and preventive strategies may include mandated baseline neurological and cognitive evaluations at the onset of an athlete’s career, with regular follow-up assessments throughout their career and into retirement, as well as regulations limiting the cumulative number of KOs an athlete may sustain. It may also be beneficial to implement structured mental health and cognitive health education programs to increase awareness of warning signs and symptoms and to help prevent maladaptive coping mechanisms. Clinically, these measures could help sports medicine professionals and athletic trainers identify athletes at higher risk for cognitive and emotional consequences and guide individualized return-to-play protocols. These approaches highlight the importance of integrating research findings into practical monitoring and policy initiatives in order to reduce long-term neuropsychological consequences in combat sports athletes.

Future research should continue to evaluate the neuropsychological effects of head trauma in MMA athletes. Subsequent studies should focus on longitudinal study designs that follow fighters through their competitive careers and into post-retirement, dose-response analysis examining the relationship between cumulative strike exposure and the severity of neuropsychological outcomes, and studies specifically focused on post-career mental health trajectories. Additionally, further work should more rigorously address the key methodological challenges within this field, including confounding variables, exposure misclassification, or survival bias as these may influence observed associations. Expanding research in these areas will strengthen evidence-based clinical decision-making and may inform the development of policies aimed at protecting athlete health.

While this review highlights emerging patterns in the literature, several limitations should be acknowledged. First, the inclusion of studies was limited to those published from 2020 onward. While this decision was made to reduce duplication of previously synthesized material, it may have excluded earlier foundational research, potentially limiting the historical scope of the analysis. Second, a formal quality appraisal of included studies was not conducted, which is consistent with scoping review methodology but limits the ability to comment on the strength and certainty of the evidence. Additional limitations include the limited number of longitudinal investigations, heterogeneity in study designs and outcome measures across combat sport populations, and the reliance of many studies on cohorts from a small number of research groups, which may restrict generalizability. The possibility of publication bias should also be considered, as studies reporting significant neuropsychological associations may be more likely to be published than null findings. Furthermore, the existing literature on neurotrauma in combat sports demonstrates an underrepresentation of female athletes, which limits the ability to generalize findings across sexes and highlights an important gap in the literature. These factors underscore the need for improved study design and the incorporation of quality appraisal tools in future reviews to strengthen the body of evidence. Additionally, screening and data extraction were conducted by a single reviewer with secondary verification, which may introduce potential bias and increase the risk that eligible articles may have been inadvertently excluded during the screening process, despite verification of the final included studies. 

Overall, the evidence that repetitive head trauma in combat sports is associated with meaningful cognitive, structural, and biomarker changes suggests potential risks for long-term neuropsychological health in this population. While the findings are derived from observational studies and vary in methodological rigor, they emphasize the practical importance of continuous monitoring, early intervention, and policy measures to protect athlete health. Future research with larger, longitudinal cohorts is needed to clarify the magnitude and trajectory of these associations and to inform targeted strategies for prevention and management.

## Conclusions

Head injuries remain a significant concern in combat sports, particularly in MMA, where athletes are frequently exposed to impacts to the head throughout their careers. While the immediate physical consequences of these injuries are often the primary focus, the literature reviewed in this paper highlights the potentially lasting neuropsychological effects associated with repetitive head trauma. Across studies, fighters demonstrate consistent vulnerabilities in cognitive domains such as processing speed, memory, and executive function, alongside evidence of structural brain changes and elevated biomarkers suggestive of neuroinflammation. These findings should be interpreted in the context of the substantial heterogeneity among the included studies with respect to participant populations, study design, outcome measures, and imaging modalities. 

These findings indicate that the burden of head trauma in combat sports may extend beyond acute injury and is associated with long-term functioning, quality of life, and post-career outcomes. Understanding the neuropsychological effects of head trauma in fighters is important for sports medicine practitioners and athletic organizations in order to help inform return-to-fight decisions, support rehabilitation strategies, and guide future safety protocols. Ultimately, improving preventive measures, detection, and management of brain injury in combat sports athletes is essential not only to protect performance, but also to preserve long-term neurological and psychological health beyond an athlete’s competitive career. Together, these findings support the value of a proactive approach that integrates objective neuropsychological assessments into clinical evaluations and regulatory standards to prioritize long-term neurological and psychological outcomes in combat sports. As the literature continues to expand, future reviews with formal quality appraisal and, where appropriate, meta-analytic methods may help further define the magnitude and consistency of these associations.
